# Radiology reporting in rectal cancer using MRI: adherence to national template for structured reporting

**DOI:** 10.1177/02841851211057276

**Published:** 2021-12-06

**Authors:** Gustav Alvfeldt, Peter Aspelin, Lennart Blomqvist, Nina Sellberg

**Affiliations:** 1 Department of Learning, Informatics, Management and Ethics, 27106 Karolinska Institutet, Stockholm, Sweden; 2 Department of Clinical Science, Intervention and Technology, 27106 Karolinska Institutet, Stockholm, Sweden; 3 Department of Molecular Medicine and Surgery, 27106 Karolinska Institutet, Stockholm, Sweden. Department of Imaging and Physiology, 59562 Karolinska University Hospital, Stockholm, Sweden

**Keywords:** Health policy and practice, staging, technical aspects, magnetic resonance imaging, rectum, structured reporting, reporting quality

## Abstract

**Background:**

In 2014, a national workshop program was initiated and a reporting template and manual for rectal cancer primary staging using magnetic resonance imaging (MRI) was introduced and made available by the national Swedish Colorectal Cancer Registry.

**Purpose:**

To evaluate the effect of the national template program by identify if there was a gap between the content in Swedish MRI reports from 2016 and the national reporting template from 2014. The aim was to explore and compare differences in content in reporting practice in different hospitals in relation to the national reporting template, with focus on: (i) identifying any implementational differences in reporting styles; and (ii) evaluating if reporting completeness vary based on such implementational differences.

**Material and Methods:**

A total of 250 MRI reports from 10 hospitals in four healthcare regions in Sweden were collected. Reports were analyzed using qualitative content analysis with a deductive thematic coding scheme based on the national reporting template.

**Results:**

Three different implemented reporting styles were identified with variations of content coverage in relation to the template: (i) standardized and structured protocol (reporting style A); (ii) standardized semi-structured free-text (reporting style B); and (iii) regular free-text (reporting style C). The relative completeness of reporting practice of rectal cancer staging in relation to the national reporting template were 92.9% for reporting style A, 77.5% for reporting style B, and 63.9% for reporting style C.

**Conclusion:**

The implementation of template-based reporting according to reporting style A is a key factor to conform to evidence-based practice for rectal cancer reporting using MRI.

## Introduction

Colorectal cancer is the third most common form of cancer in Sweden. About 2100 persons are diagnosed with rectal cancer in Sweden annually (1). The advancement of different anti-cancer treatment options has driven the need for individualized cancer management (2). Primary staging and restaging of rectal cancer after neoadjuvant treatment are essential parts of the cancer treatment management for best possible patient outcome (3,4). Magnetic resonance imaging (MRI) is considered the first-line imaging modality for primary staging and restaging of rectal cancer (5) and several studies confirms the importance of the key prognostic elements when interpreting and reporting findings (3,6–9).

Template-based reporting using a standardized proforma or protocol is a potential way to increase the staging accuracy (10,11). The importance of proper reporting is also emphasized by the expert consensus panel of the European Society of Gastrointestinal and Abdominal Radiology, who states that rectal cancer staging should be “reported in a structured fashion so that important findings impacting directly on therapeutic decision making are not omitted” (8, pp. 2523).

Problems related to lack of reporting unity and the need for a more standardized and structured alternative, whether it has been termed structured reporting, template-/protocol-/proforma-based reporting, itemized reporting, or synoptic reporting, has been described for over a decade, both from radiologists and pathologists as well as their clinical counterparts (12–15).

By 2014, the Swedish Colorectal Cancer Registry (SCRCR), together with members from the Swedish Society of Radiology (SFMR) had developed a national radiology reporting template and related instruction manual for primary staging of rectal cancer (16,17) that was introduced via a national workshop program. These were based on the ESMO clinical practice guidelines for rectal cancer (18) and the work of the MERCURY study group (3,6,19), and was designed to withhold the, by the time available, key prognostic elements for rectal cancer staging, with a strong link to the SCRCR protocol for reporting of findings to the national registry (20). This was a clear initiative to improve radiological practice in the field but besides registering the completeness of reporting of rectal cancer to the national registry, no evaluation of the effect of this initiative has been performed.

The aim of the present study was to evaluate the effect of the national template initiative by identifying a potential gap between the content in Swedish MRI reports created in 2016 and the national reporting template from 2014. The secondary aim was to explore and compare differences in content in reporting practice in different hospitals in relation to the national reporting template, with a focus on: (i) identifying any implementational differences in reporting styles; and (ii) evaluating if reporting completeness vary based on such implementational differences.

## Material and Methods

### Sample size and filtering of radiology reports

The unit of analysis in this study were rectal cancer primary staging reports authored in 2016, two years after the introduction of the national reporting template, collected from 10 hospitals in four healthcare regions in Sweden—the three largest regions, and one smaller region (Table 1). The study protocol was vetted and approved by the Swedish Ethical Review Authority (Ethics approval number: 2017/695-31/5). Patient reports were identified via the SCRCR and assorted by hospital and radiology department. The healthcare regions and the hospitals were chosen based on the number of patients with rectal cancer at each site. Regions and hospitals with a high number of patients reported in SCRCR were asked to contribute with data. Each radiology department provided de-identified reports from their reporting system, filtered by the first pelvic MRI after diagnosis before treatment. Since the study was conducted retrospectively from de-identified data, the Swedish Ethical Review Authority determined that the study did not need to obtain patient informed consent.

**Table 1.** Statistical overview of the reports at each hospital.

Healthcare region	Hospital	Total reports	Compliant reports	Selected reports
Region A	Hospital 2	147	38	25
	Hospital 3	113	67	25
	Hospital 8	101	55	25
Region B	Hospital 9	194	115	25
	Hospital 5	74	42	25
	Hospital 1	247	51	25
Region C	Hospital 10	116	66	25
	Hospital 4	74	50	25
	Hospital 7	76	47	25
Region D	Hospital 6	61	36	25
	Total	1203	567	250

A total of 1203 reports were obtained. Due to issues with non-standardized reporting systems and lack of standardized information models and terminologies, neither of the healthcare regions could filter out only the reports that were of interest to this study. Thus, several exclusion criteria needed to be applied manually on the given reports (Table 2). After applying the listed exclusion criteria, 567 reports were found compliant to this study (Table 1).

**Table 2.** Exclusion criteria for MRI reports.

No.	Exclusion criteria	Comment
1	Demonstration and/or multidisciplinary conference reports	These reports already have a previous “original” staging report
2	Reports written other year than 2016	This could happen since year of diagnoses does not always equal year of exam
3	Reports written by subcontractors and not the actual hospital radiology department	I.e. referrals that were forwarded to, and answered by, another radiology department
4	Reports where the examined body part is something else than the abdominal region (abdomen, lower abdomen, pelvis, rectum)	Combinatory procedures are included if they combine abdominal MRI with some other procedure, e.g. liver CT for review of metastases
5	Reports where the modality is something else than a pelvic MRI	E.g. if the procedure is an abdominal CT
6	Non-staging exams	
7	Follow-up exams	E.g. a staging exam after neoadjuvant treatment
8	Cancelled exams	Such exams typically end up with a final report stating that the exam was cancelled in the observations or findings section
9	Exams without rectal tumour findings	E.g. if the reason for referral states a rectal cancer staging but the radiology findings does not concur, there is a tumour to stage
10	Exams where the “reason for study” equals “status post op” or “relapse”	
11	Other kind of tumour findings	E.g. anal/colon cancer and not rectal cancer
12	Duplicates	Some datasets contained duplicates of reports

CT, computed tomography; MRI, magnetic resonance imaging.

The datasets were staged using Microsoft Excel version 2016 before being imported into QSR International Nvivo 11 for coding and analysis. The staging phase involved steps and measures in harmonizing the datasets from the different hospitals. The 567 compliant reports were randomized in Microsoft Excel using the RAND-function. A total of 25 randomized reports from each hospital were imported into Nvivo. All 250 reports were considered adequate to reach data saturation and information power (21,22) to make valid inferences about the content in communicated rectal cancer primary staging reports.

### Annotating MRI reports using content analysis

The radiology reports were interpreted and coded using a deductive content analysis research technique by means of coding the report contents to predefined categories in a coding scheme with a thematic approach, i.e. dividing the content of the reports into shorter units or segments with shared thematic meaning (23–27).

**Fig. 1.**
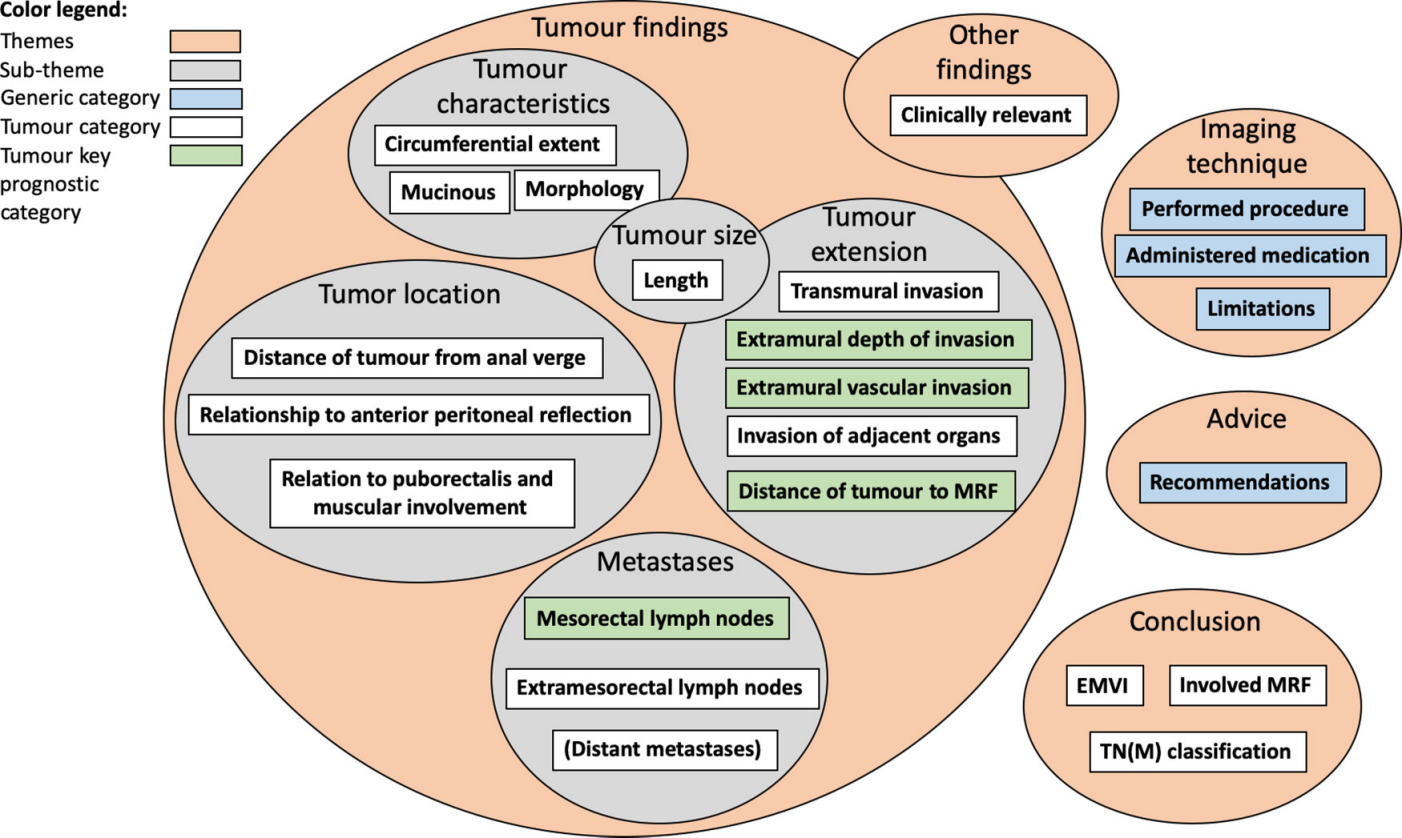
Visualization of predefined coding scheme.

The predefined coding scheme was created consisting of: (i) themes; (ii) sub-themes; and (iii) categories. The 18 tumour-specific categories and sub-themes were based on the national reporting template for primary rectal cancer staging (16) and the corresponding SCRCR protocol (20) with a structure of themes and generic categories conforming to established radiology practice guidelines (28,29). Of the 18 tumour-specific categories, four were considered as key findings of pathological prognostic importance (7). A visualization of the predefined coding scheme is illustrated in Fig. 1.

The unit of text to be coded, the recording unit, can be coded to the same theme and category if it shares the same semantics (24,25,30). Examples of coded recording units is presented in Tables 3 and 4. Recording units that could not be coded to any of the predefined categories were coded to a temporary category and later analyzed by an abdominal radiologist expert (LB) to determine if they belonged to an existing category or a new category. In this way, new categories can be introduced like in an inductive approach (24,31,32).

**Table 3.** Examples of recording units and how they are coded to a category within a theme.

No.	Recording unit	Category	Sub-theme	Theme
1	The tumour covers about three-quarters of the circumference with the main growth dorsally and to the left	Circumferential extent	Tumour characteristics	Tumour findings
2	[discontinuity of the muscularis propria] with smaller (1–5 mm) tumour extensions into the MRF	Extramural depth of invasion (EMD)	Tumour extension	
3	The shortest distance of the tumour to the MRF is 3 mm	Distance of tumour to MRF (CRM)	Tumour extension	
4	The tumour is situated in level with and below the anterior peritoneal reflection	Relationship to anterior peritoneal reflection	Tumour location	
4	In conclusion, a low tumour estimated as T3b/T4b, N1	TN(M) classification	-	Conclusion

Content within square brackets are contextual notes written by the authors to facilitate reading of recording units which has been parted from longer sentences.

EMD, extramural depth of invasion; MRF, mesorectal fascia.

**Table 4.** Examples of content comparison of recording units within the same category between type A styled reports, type B styled reports, and free–text-based reports.

No.	Recording unit	Hospital	Reporting style	Category
1	18. The tumour ends 135 mm from anal verge	Hospital 10	A	Distance of tumour from anal verge
2	18. The distance between the anal verge and the lowest part of the tumour: 30 mm	Hospital 9		
3	18. The distance between the anal verge and the lowest part of the tumour is about 7 cm	Hospital 9		
4	18: 3.5 cm	Hospital 9		
5	[a tumour] beginning 7 cm from the anal…	Hospital 6	B	
9	[a tumour] with the lowest part 4 cm from anal verge	Hospital 7		
12	Beginning approximately 4 cm above anal verge [there is a tumour]	Hospital 4	C	
13	7 cm from anus in oral direction [there is a tumour]	Hospital 4		
14	About 135 mm proximal to anus [there is a tumour]	Hospital 4		

Content within square brackets are contextual notes written by the authors to facilitate reading of recording units which has been parted from longer sentences.

By applying this approach on the total cohort of reports, all the recording units of the reporting content have been allocated to a specific theme and category, predefined or new, based on its semantics. If no findings were to be omitted according to what was being mentioned in the national reporting template, each report would consist of recording units coded to all 18 predefined tumour categories, giving a total number of at least 450 recording units at each of the 10 hospitals.

Due to the risk that reports can contain errors and that descriptions of findings can be ambiguous and difficult to interpret on account of subjective reasoning and underlying meanings (12,33–37), a computer-aided approach to content categorization has not been chosen for this study as it might reproduce any mistakes if they existed.

### Clinical relevance and trustworthiness

The main issues in content analysis relates to the concept of trustworthiness (25–27). Since each individual has their own pre-understanding and is prone to interpret data accordingly, there is always the risk of coding issues, alternative interpretations, or misinterpretations (24). To minimize such risks and to achieve as high an intercoder reliability and accuracy as possible in the coding process, after the initial coding was performed the coding was double-checked by an abdominal radiologist (LB) and randomly selected coding was spot checked by another radiologist (PA). Some texts were also coded more than one time to ensure coding consistency.

## Results

**Fig. 2.**
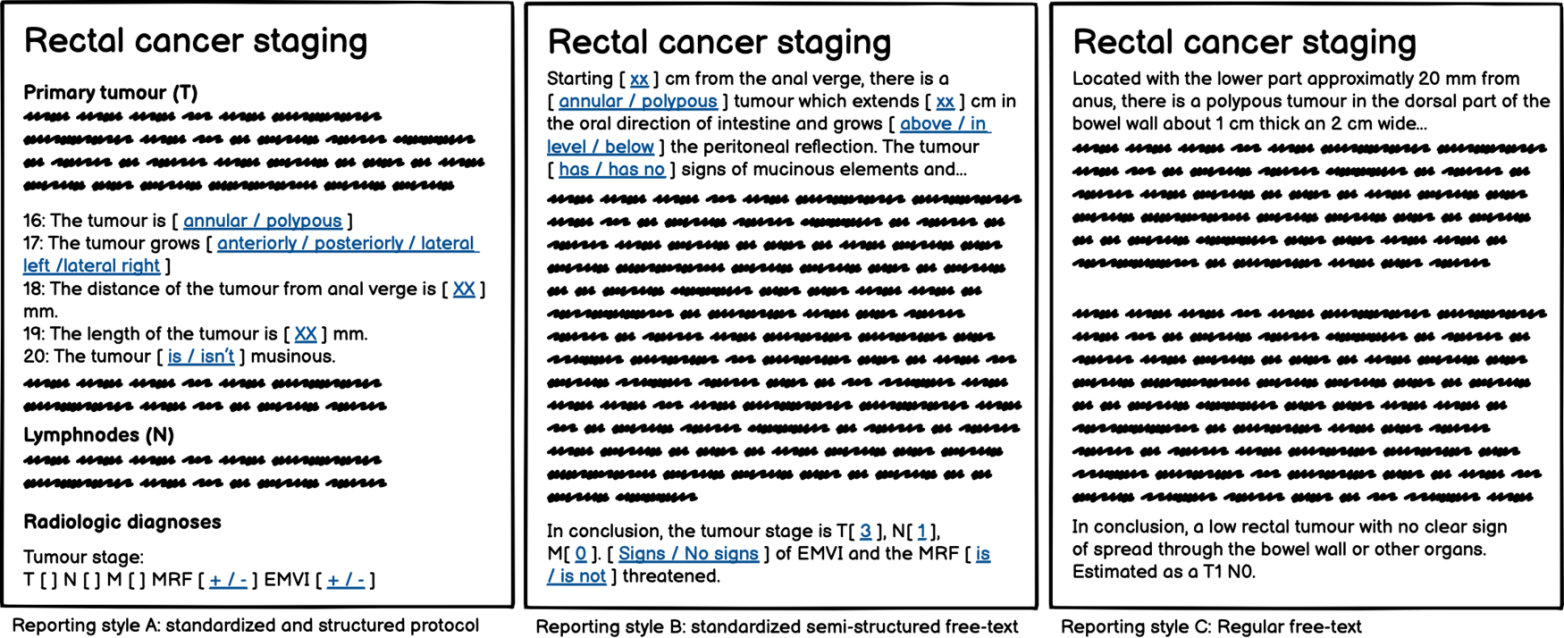
Examples of reporting styles A, B, and C.

**Fig. 3.**
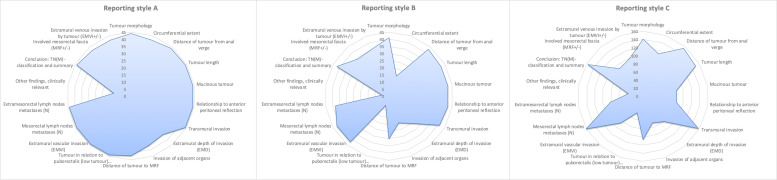
Recording units per reporting style in correlation to the national reporting template and the predefined tumour-specific categories.

**Fig. 4.**
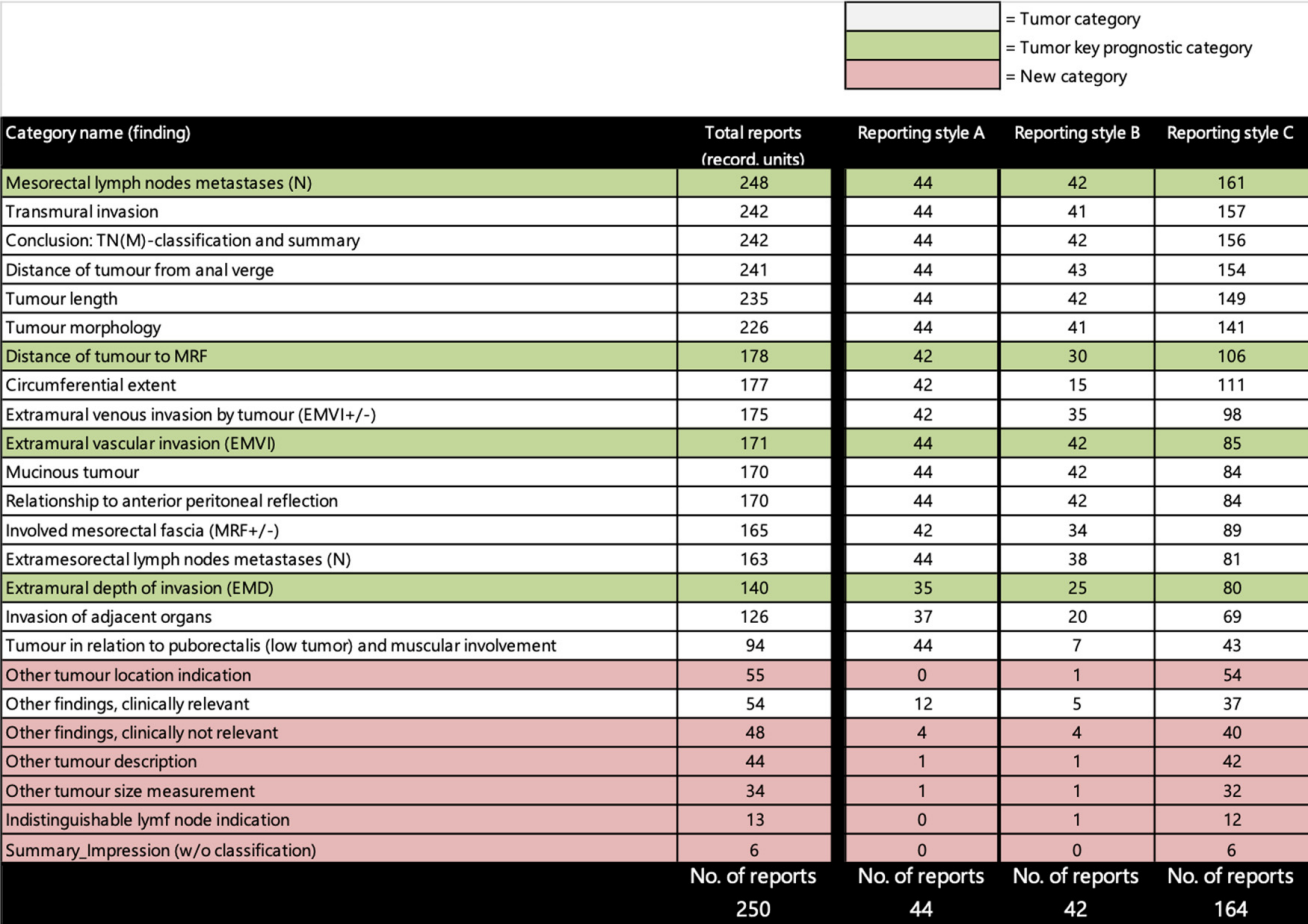
Result of coded recording units to categories. The figure shows the categories sorted based on the total report count of recording units, the predefined categories including the categories with key findings of pathological prognostic importance. The new categories that were not part of the predefined coding scheme, but created during the coding process, are also presented.

Three different types of reporting styles were identified, with varying degrees of content coverage, information completeness, and harmonized terminology in relation to the content of the national reporting template. We divided the three reporting styles as follows: A = reports using a standardized and structured reporting protocol; B = reports using a preconfigured, semi-structured free text; and C = regular free-text reports. Reporting styles A and B are both variations of template-based reporting. In style A, there is a strong link to the national reporting template. Style B was defined by clear signs of a preconfigured template with semi-structured free text, i.e. with more or less the same findings being reported, in the same order and with similar terminology. The link to the national reporting template were not as pronounced as in style A, with not all of the findings being reported. Style C means that there were no signs of any repetitiveness in either the structure, the number of findings being reported, or the use of terminology. Examples of reporting styles A, B, and C are shown as mockups (Fig. 2). The content coverage of the three reporting styles A, B, and C are graphically illustrated in radar charts (Fig. 3) and numerically shown in Fig. 4.

**Fig. 5.**
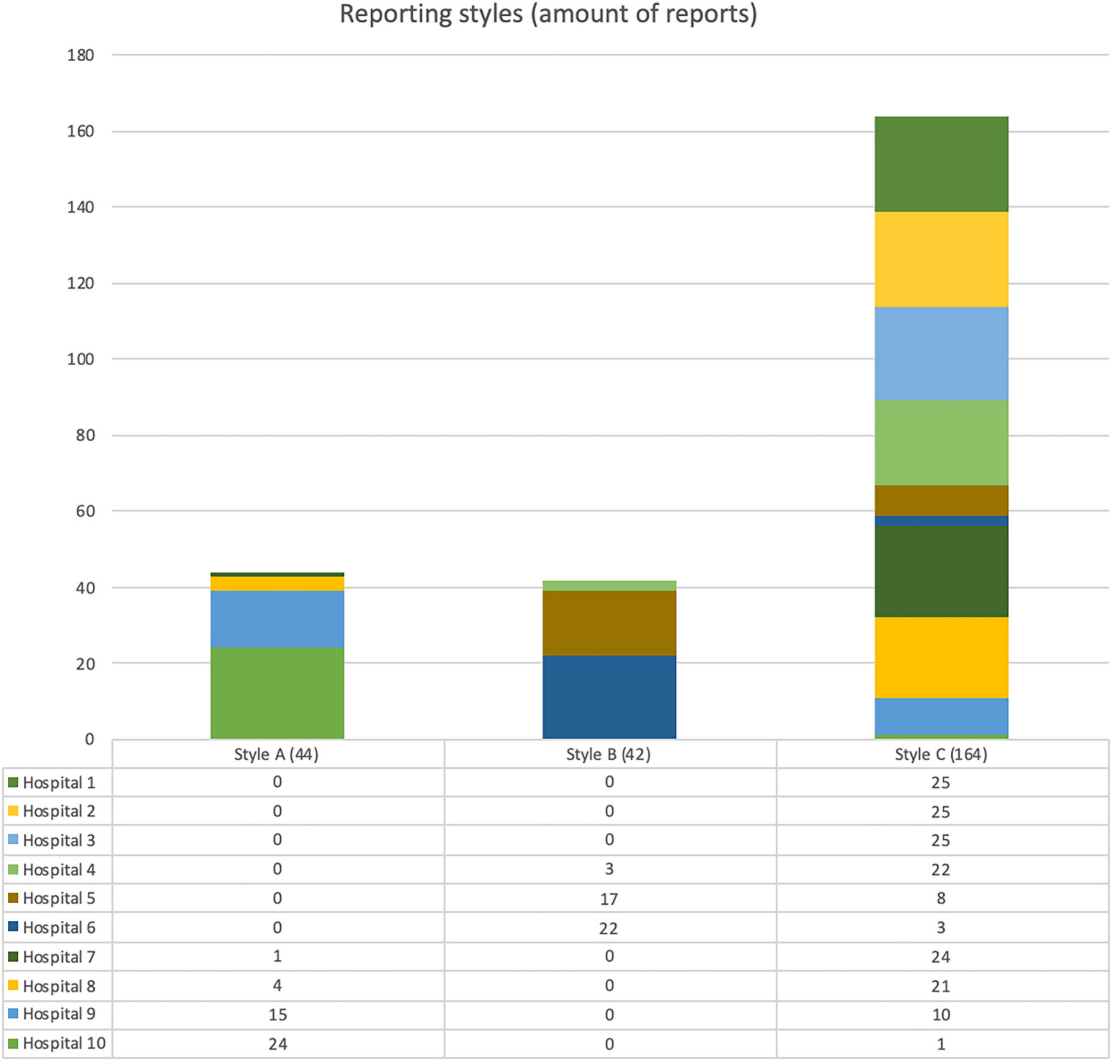
Total amount of reports per reporting style and hospital.

Of the 250 reports, 86 were based on a template. Of these, 44 were conformant to reporting style A and 42 to style B. The rest of the 164 reports were conformant to reporting style C. Two hospitals were highly compliant to the national reporting template. Two hospitals were using style B and six hospitals were showcasing a mix with predominantly style C but individual occasions of style A or B (Fig. 5).

### Reporting style A: standardized and structured protocol

There was nearly no gap between the national reporting template and reporting practice when using reporting style A; 99.2% of the reported content correlated with the national reporting template and the predefined tumour-specific categories of the coding scheme. With the adjustment for omitted information, the relative completeness was 92.9%. This gives an average of about 16.7 out of 18 predefined tumour-specific findings per report. As can be seen graphically in Fig. 3, the only category with an obvious dip of coded recording units was the category of clinically relevant “Other findings.” However, it is also notable that some reports omitted extramural depth of invasion (EMD) despite being a key finding. Almost no findings other than the ones mentioned in the national reporting template were being reported when using reporting style A (Fig. 4).

### Reporting style B: semi-structured free text

There was a larger gap between the national reporting template and everyday reporting practice of rectal cancer primary staging when using type B style reporting than using reporting style A. Of all reported content, 98.7% correlated with the national reporting template and the predefined tumour-specific categories of the coding scheme. However, making adjustment for omitted information, the relative completeness dropped to 77.5%. This gives an average of about 14 out of 18 predefined tumour-specific findings per report. Reporting style B demonstrates more categories with visible dips of coded recording units, beyond the category of clinically relevant “Other findings,” in comparison to reporting style A. Most notable are the omitted key findings EMD and distance to mesorectal fascia (Fig. 3). As in reporting style A, almost no findings other than the ones mentioned in the national reporting template were being reported when using reporting style B (Fig. 4).

### Reporting style C: regular free text

Hospitals with reporting style C hade the largest gap between reporting practice and the national reporting template. Of all reported content, 91.0% correlated with the national reporting template and the predefined tumour-specific categories of the coding scheme. However, adjusting for omitted information, the relative completeness dropped to 63.9%. This gives an average of about 11.49 out of 18 predefined tumour-specific findings per report. Reporting style C had more categories with lower amount of coded recording units and noticeable fewer peak categories than the other reporting styles (Fig. 3). Also, the free–text-based reports were not as stringent as the template-based reports of styles A and B and contained more findings beyond than the ones mentioned in the national reporting template (Fig. 4).

## Discussion

The present study is based on a qualitative content analysis of MRI reports for rectal cancer staging authored at 10 hospitals in Sweden in 2016 by utilizing a predefined thematic coding scheme based on the national reporting template and related instruction manual for rectal cancer primary staging that was introduced by SCRCR in 2014 (16,17).

We found three distinctive types of implemented reporting styles: A = standardized and structured protocol; B = standardized semi-structured free text; and C = regular free text.

The different reporting styles show variations of content coverage in relation to the national reporting template. The relative completeness of everyday reporting practice of rectal cancer staging in relation to the template ranged between 92.9% (A) and 63.9% (C), meaning there was a ranging gap of 7.1%–36.1% between the content of the template and the reporting practice based on differences in reporting style.

Total conformance to the national reporting template of rectal cancer staging would mean 100% information completeness and would also contribute to the use of an agreed harmonized terminology. Information completeness, conformance to current standards, consistency, and clarity of communication are all considered key parameters in reporting quality (34,38). This is being confirmed by the template-based reporting of reporting style A, where almost all the findings mentioned in the template were accounted for in the reports in a consistent way.

However, even if conformant to a template, the reporting completeness was significantly less, and the content variety was more widespread in reporting style B in comparison to style A. By using a template with a preconfigured standardized text, we noticed gaps among the different findings. If the author of such a template considers some type of finding irrelevant, it can affect many reports by excluding information of a specific type of finding. The latter is also an example of limitation when it comes to negated information, is it important to express the absence of a finding or not? In Figs. 3 and 4, we show that the mentioning of a low tumour finding is complete when it comes to reports using reporting style A. This is not because all these reports consist of low tumours, but the fact that the reports mention the negated finding. Nonetheless, the completeness and consistency in reporting style B are an improvement in comparison to traditional free-text reporting as seen in reporting style C.

Regardless of type A or type B styled reports, differences in comparison between the hospitals and within a single hospital can be seen. It can relate to variances in terms of units, wordings, and medical terminology, e.g. the units can vary within one radiology department signaling internal implementational differences even when using reporting style A (Table 4). In some cases of type A styled reporting, the context of the findings was disregarded as exemplified in row 4 of Table 4, where there is a need for the reader to be familiar with the variables of the national template to be able to understand the context of the measurement. Variations in style A and B reports, despite being template-based, may be explained by authors editing certain parts of the template if some findings are considered insignificant or missing.

Apart from reports conforming to the template-based reporting styles of A and B, the overall completeness of free-text reporting in accordance to reporting style C demonstrates knowledge of the findings mentioned in the national reporting template. In a previous article, we identified that the relative completeness of the reported content were 48% for free-text reports authored before the introduction of the national reporting template (39). In reporting style C, 63.9% of the findings mentioned in the template could be accounted for but not in a recurring, standardized, or structured way. It is significant for the reports of reporting style C that they contain more information related to the new categories of “Other” than the template-based reports of reporting styles A and B. As can be seen in Fig. 4, the overall coding of recording units to any of the new categories were 200 instances on a total. The type C styled free-text reports contained 186 of these.

Even if there is progress regarding the content of the free-text reports of reporting style C, studies within the field of reporting quality and structured reporting have pointed out that the satisfaction rate of free-text reporting can be as low as 40% (40). Template-based reports have a higher occurrence of key tumour descriptors than free-text reports (41,42) as well as a higher frequency of correct tumour T and N stage (10). The pattern seems to be the same in other diagnostic domains that also shows the importance of standardized datasets to ensure reporting quality (38).

The introduction of the national reporting template and manual for rectal cancer staging in 2014 (16) had positive effects regarding reporting completeness. There are some strong arguments in favor of template-based reporting, especially in the form of reporting style A with a reduction of reporting differences between healthcare organizations and increased reporting quality. Even though certain key components are still missing in some of the template-based reports, this is a strong signal for a shift in how reporting is done. There are still challenges regarding the implementation of standardized and structured templates into the different reporting systems and the organizational complexities around professional agreement when it comes to manage a template, regardless of whether it is a local, regional, or national template.

Template-based reporting as seen in reporting style A is a first step towards a higher reporting quality in terms of mentioned quality parameters. Nonetheless, it is still far from the more advanced levels of structured reporting implementation as described by Ellis et al. (38), with agreed value sets, discrete data, and terminology binding to established terminologies or ontologies such as SNOMED CT (43,44).

Higher levels of structured reporting also relate to the discussion of computer-aided diagnostics, deep learning algorithms, and other types of automated processes and secondary uses such as reporting to registries, research, or other kinds of follow-ups that might have use for a more structured information (45,46).

A limitation of this study, apart from the known issues associated with qualitative content analysis, is the fact that the data analyzed is five years old. Even if the purpose of this study was to evaluate the effect of a national template program, two years after its introduction, and to explore and compare differences in content in reporting practice in different hospitals in relation to a recently presented national reporting template, reporting styles could have transformed in the amount of time since then and the results, both with regard to content and structure, might not be transferable to radiology reporting practice in 2021.

For future research, it would be interesting do a deeper investigation to identify critical barriers to the implementation of a more structured and uniform reporting with lesser variations that could be used for targeted initiatives overcoming these barriers.

In conclusion, reporting practice supported by template-based reporting supersedes free-text reporting by far when it comes to information completeness, harmonized terminology, and standardized and structured content. We postulate that the implementation of template-based reporting in the reporting systems is a key factor to conform to evidence-based practice.

## Data Availability

The datasets used and analyzed for this study are available from the corresponding author on reasonable request
